# Clinical efficacy of Gualou Xiaoyong Decoction and painless lactation manipulation in treating lactation acute mastitis and breast abscess: An observational study

**DOI:** 10.1097/MD.0000000000034617

**Published:** 2023-08-25

**Authors:** Huijun Ye, Dexin Hu, Huiling Zheng, Yi Yang, Yunxia Lin, Jiali Liu, Xi Luo, Ruilan Li, Fengying Hu, Lihua Jin

**Affiliations:** a Department of Obstetrics and Gynecology, The Second Affiliated Hospital of Zhejiang Chinese Medical University, Xinhua Hospital of Zhejiang Province, Hangzhou, China; b Department of Orthopaedics, Hangzhou Red Cross Hospital, Hangzhou, China.

**Keywords:** breast abscess, clinical efficacy, Gualou Xiaoyong Decoction, lactation acute mastitis, painless lactation manipulation, retrospective analysis

## Abstract

Information on the effects of Chinese medicine in the treatment of lactational acute mastitis and breast abscess is limited; thus, we conducted an observational study to analyze the clinical efficacy of Gualou Xiaoyong Decoction combined with painless lactation manipulation in the treatment of lactational acute mastitis and breast abscess. A total of 41 patients with lactational acute mastitis and breast abscess who were treated with Gualou Xiaoyong Decoction and painless lactation manipulation from October 2021 to October 2022 were included in this study. The age, fetal times(primiparous/multiparous), delivery mode (cesarean section/vaginal delivery), onset time, breast lump diameter, skin rash diameter, body temperature, visual analogue score, blood routine, C-reactive protein, procalcitonin, bacterial culture in milk, B ultrasound and other data of these patients were statistically analyzed. After treatment, the breast lump diameter of these patients decreased significantly, the skin rash diameter was reduced or disappeared, the body temperature decreased or returned to a normal range, and the visual analogue score also decreased. Besides, these patients had a decreased total number of white blood cells and a reduced percentage of neutrophils, C-reactive protein, and procalcitonin after treatment. In addition, bacteria in the milk of most patients disappeared, and there was no abnormality in B ultrasonic imaging. Except for 2 patients with breast abscess who stopped breastfeeding on the affected side for 1 day and 3 days respectively, all other patients continued to provide breast milk for their infants, and no adverse reactions were observed in these infants. The combination of Gualou Xiaoyong Decoction and painless lactation manipulation can achieve favorable clinical effects in the treatment of lactational acute mastitis and breast abscess. This combined therapy has good efficacy, short course of treatment, low costs, and great convenience with the avoidance of pain, hospitalization, influence on lactation, breast scar and other adverse outcomes.

## 1. Introduction

The World Health Organization (WHO) encourages exclusive breastfeeding for the first 6 months of life. However, the global exclusive breastfeeding rate for infants aged 0 to 6 months is only 43%, and only 29.2% of infants aged 0 to 6 months in China receive exclusive breastfeeding.^[1]^ Among these mothers during lactation, acute mastitis and breast abscess are considered to be the leading causes of accidental weaning. In particular, patients with breast abscess often stop breastfeeding (41%) or suffer from breast fistula (11.1%) and readmission (50%).^[2,3]^ Mammitis during lactation is a common and prevalent clinical disease, and it is an acute suppurative inflammation in the mammary duct and surrounding connective tissue.^[4]^ It is characterized by acinar injury, edema, and inflammatory cell infiltration of the affected breast.^[5]^ This disease may cause local pain in the affected breast, and it is often accompanied by rapid systemic symptoms, including fever, muscle pain, chills, fatigue, and other flu-like symptoms.^[2]^ In severe cases, the affected breast may be permanently disfigured. According to statistics, about 33% of mothers during lactation have episodic history of acute mastitis,^[6]^ among whom 4.0% to 8.5% have a history of recurrent episodes.^[4]^ In case of improper treatment, it may lead to breast abscesses, breast fistula or even sepsis, septic shock, and chronic inflammation in the breast that persists or has multiple acute attacks.^[5]^ In the previous, lactation acute mastitis and breast abscesses were often treated by intravenous infusion or oral antibiotics, and breast puncture and surgical incision and drainage would be often adopted too. However, these methods are often limited by unfavorable efficacy, long treatment course, requirements for stopping breastfeeding, trauma, patient suffering, high possibility to produce antibiotic-resistant strains, chronic breast lumps, and reduced maternal milk. In this study, Gualou Xiaoyong Decoction combined with painless lactation manipulation was applied to treat lactational acute mastitis and breast abscesses and achieved favorable clinical effects. The clinical data of these patients were retrospectively analyzed.

## 2. Data and methods

### 2.1. Case source

#### 2.1.1. Diagnostic criteria of acute mastitis during lactation.

Acute mastitis during lactation can be diagnosed based on the following criteria: (1) breast lump, pain, and local redness, with or without increased skin temperature; (2) systemic inflammatory reactions, such as chills, headache, and fatigue; (3) body temperature ≥ 37.3 °C; or increased white blood cell (WBC), neutrophils (NE), or C-reactive protein (CRP) in blood routine examinations; (4) positive milk bacterial culture.^[7]^

#### 2.1.2. Diagnostic criteria for breast abscess during lactation.

In addition to the diagnostic criteria of lactational mastitis, the following inclusion criteria shall also be met: (1) no echo area or low echo area in the ultrasonic examination, and the presence of fluid flow after pressurization; or/and (2) the discharge of pus.^[1]^

### 2.2. Treatment methods

#### 2.2.1. Treatment based on traditional Chinese medicine.

Gualou Xiaoyong Decoction was composed of Chaihu (9 g), Gualou peel (15 g), Luffa (15 g), Lobelia (15 g), Dandelion (15 g), Paeonia Rubra (15 g), Tongcao (6 g), Peach Kernel (10 g), Fried Coix Seed (30 g), Chixiaodou (20 g), Angelica (15 g), Fried Atractylodes macrocephala (15 g), Arctium lappa (15 g), Honeysuckle (20 g), and Forsythia suspense (15 g). Specifically, 400 mL of the medicinal solution was decocted for oral administration in the morning and afternoon, with 200 mL in the morning and afternoon, respectively (one dose each day and a total of 5 doses).

#### 2.2.2. Painless lactation manipulation.

The points were selected along the channels and perform local breast massage once a day. The lactation manipulation can be discontinued after the resolution of breast lumps.

① Point selected along the channels: Tanzhong, Ruzhong, Rugen, Qimen, Shaohai, Chize, Shaoze, Tianchi, Tianxi, etc. Each acupuncture point was pressed for 1 to 2 minutes.

② Local breast massage: Place the thumb and index finger of one hand at the junction of black and white areola skin. Subsequently, remove the front milk with the downward and inward pressing method. Then, apply sesame oil or olive oil and other media on the hand, gently massage the breast lump 3 times with the finger pulp or thenar to discharge the milk in the direction of the nipple, and massage each breast for 20 to 30 minutes with this technique.

### 2.3. Observation indexes

The body temperature, visual analogue score, breast lump diameter, skin rash diameter, blood routine examination (mainly including WBC count and the percentage of NE [NE%]), CRP, procalcitonin (PCT), milk bacterial culture, and B ultrasound of these patients were examined before and after 5 days treatment. During statistics, patients with a body temperature ≥ 37.3 °C would be regarded as positive, while those with a body temperature < 37.3 °C would be regarded as negative. Additionally, patients with abnormal ultrasound (irregular hypoechoic area or anechoic area) would be classified as positive, while those without obvious abnormality (normal ultrasound image) would be classified as negative.

### 2.4. Bacterial culture method of milk

After the affected nipple was disinfected with the povidone iodine solution, it was wiped dry with a disinfection cotton swab. Then, a qualified nurse squeezed the superficial milk. Finally, the deep milk was collected into a bacterial culture tube and sent to the bacterial laboratory for bacterial culture.

### 2.5. Inclusion criteria

Patients who met the diagnostic criteria of acute mastitis and breast abscess during lactation and had complete medical records would be included in this study. This study was approved by the Medical Ethics Committee of the Second Affiliated Hospital of Zhejiang University of Traditional Chinese Medicine [Approval No. 2022-137-001].

### 2.6. Exclusion criteria

Patients with granulomatous mastitis, incomplete medical records, incomplete laboratory data, and loss to follow-up would be excluded.

### 2.7. Clinical efficacy criteria

Cure: General symptoms disappeared and the breast lump was resolved. Improvement: Systemic symptoms disappeared, and local swelling and pain were relieved. Ineffective: There was no mitigation in symptoms, with repeated cyst transmission or the formation of breast fistula.^[8]^

### 2.8. Statistical methods

SPSS 22.0 was used to conduct a statistical analysis. The mean measurement data were compared with the T test, and the measurement data before and after treatment were compared with the paired T test. The counting data rate was compared with the χ2 test. *P* < .05 indicated that there was statistical significance.

## 3. Results

### 3.1. Basic information (n = 41)

See Table 1 for details.

**Table 1:** Basic information of maternity.

Basic information	Value
Average age (years)	30.93 ± 2.81
Average parities (times)	1.17 ± 0.38
Average postpartum onset time (days)	83.56 ± 95.00

### 3.2. Delivery mode

See Table 2 for details.

**Table 2:** Maternal delivery mode.

Delivery mode	Number	Proportion (%)
Vaginal delivery	30	73.2%
Cesarean section	11	26.8%

### 3.3. Parity (primiparous/multiparous)

See Table 3 for details.

**Table 3:** Parity (primiparous/multiparous).

Primipara (yes/no)	Cases	Proportion (%)
Yes	34	82.93%
No	7	17.07%

### 3.4. Changes of clinical symptoms before and after treatment

After treatment, the breast mass diameter decreased significantly, the skin rash diameter was reduced or disappeared, the body temperature decreased or returned to a normal range, and visual analogue score decreased significantly (*P* < .01). (Table 4).

**Table 4:** Changes of clinical symptoms before and after treatment.

Clinical symptom indicators	Pre-treatment	Post-treatment	*P*-value
Maximum mass diameter	7.20 ± 3.44	1.24 ± 2.06	<.01
Maximum diameter of rash	3.49 ± 3.95	0.24 ± 1.56	<.01
Temperature	37.61 ± 1.13	36.53 ± 0.32	<.01
Number of people with positive body temperature	20/41 (48.8%)	1/41 (2.4%)	<.01
VAS	6.51 ± 2.01	0.51 ± 1.05	<.01

### 3.5. Changes of laboratory indexes before and after treatment

Patients had a significantly decreased total number of WBC and a significantly reduced percentage of NE, CRP, and PCT (*P* < .01). The bacteria in the milk of most patients disappeared, and there was no abnormality in B ultrasonic imaging. (Table 5).

**Table 5:** Changes of laboratory indexes before and after treatment.

Laboratory indicators	Pre-treatment	Post-treatment	*P*-value
Total number of white blood cells (10^9^/L)	10.47 ± 4.30	6.80 ± 1.99	<.01
Percentage of neutrophils (%)	72.09 ± 12.34	57.39 ± 10.78	<.01
C-reactive protein (mg/L)	38.25 ± 38.20	14.27 ± 17.33	<.01
PCT (mg/L)	0.06 ± 0.05	0.04 ± 0.05	<.01
Patients with positive bacterial culture	17/41 (41.46%)	7/41 (17.07%)	<.05
Patients with positive B-ultrasound	14/41 (34.15%)	2/41 (4.88%)	<.01

### 3.6. Results of milk bacterial culture before and after treatment

All patients received milk bacterial culture before and after treatment. Among them, 17 patients had positive bacterial culture before treatment, and 7 patients had positive bacterial culture after treatment. After 5 days of treatment, the milk was collected again for bacterial culture. (The result would be considered negative if there was no growth of bacteria and fungi after 48 hours of culture.) (The negative rate (%) = The number of negative cases/The number of positive cases before treatment * 100%.)

It can be seen from the above diagrams (Table 6) that there are various pathogenic bacteria in the milk from the affected breast, which can be infected by one bacterium alone or by multiple bacteria together. Staphylococcus is the main pathogen. However, the pathogenic staphylococcus family contains many members, including *Staphylococcus aureus, Staphylococcus epidermidis, Staphylococcus warneri, Staphylococcus tardus*, and *Staphylococcus hominis*. In addition, there are other streptococcus families in the bacterial culture, such as *Viridans streptococci* and salivary streptococcus. The types of bacteria in the milk may change after treatment. The culture results of most bacteria in the milk become negative after Gualou Xiaoyong Decoction in combination with painless lactation manipulation.

**Table 6:** Results of milk culture before and after treatment.

Bacteria in milk	Pre-treatment (cases)	Post-treatment (cases)	Negative conversion rate (%)
*Staphylococcus aureus*	4	2	50
*Staphylococcus epidermidis*	6	5	16.67
*Staphylococcus warneri*	2	0	100
*Staphylococcus tardus*	1	0	100
*Staphylococcus homini*s	1	0	100
*Streptococcus salivarius*	1	0	100
*Streptococcus viridis* + *Staphylococcus aureus*	1	0	100
No bacterial growth	24	24	/

As shown in Table 7, the results of 13 patients with positive bacterial culture before treatment become negative after treatment, with a negative rate of 13/17 (76.47%). One patient with salivary streptococci in the milk bacterial culture develops Staphylococcus epidermidis after treatment, and 3 patients without bacteria in the milk bacterial culture before treatment develop *Staphylococcus epidermidis* after 5 days of treatment.

**Table 7:** Changes of patients with abnormal bacterial culture before and after treatment.

Medical record no.	Pre-treatment	Post-treatment
7	*Staphylococcus epidermidis*	No bacterial growth
8	*Staphylococcus warneri*	No bacterial growth
16	*Staphylococcus warneri*	No bacterial growth
17	*Staphylococcus epidermidis*	No bacterial growth
18	*Streptococcus salivarius*	*Staphylococcus epidermidis*
20	*Staphylococcus aureus*	No bacterial growth
21	*Staphylococcus aureus*	No bacterial growth
22	*Staphylococcus epidermidis*	No bacterial growth
23	No bacterial growth	*Staphylococcus epidermidis*
25	*Streptococcus viridis, Staphylococcus aureus*	No bacterial growth
27	*Staphylococcus epidermidis*	No bacterial growth
28	*Staphylococcus tardus*	No bacterial growth
29	*Staphylococcus hominis*	No bacterial growth
31	*Staphylococcus epidermidis*	No bacterial growth
32	No bacterial growth	*Staphylococcus epidermidis*
33	No bacterial growth	*Staphylococcus epidermidis*
35	*Staphylococcus epidermidis*	*Staphylococcus epidermidis*
39	*Staphylococcus epidermidis*	No bacterial growth
40	*Staphylococcus aureus*	*Staphylococcus aureus*
41	*Staphylococcus aureus*	*Staphylococcus aureus*

### 3.7. Breastfeeding

Breastfeeding was recommended for all patients with mastitis during lactation, and there were no such adverse reactions as crying, diarrhea, abdominal distension, nausea, vomiting, fever, and cough in infants. As listed in Table 8, among 3 patients with breast abscess, pus can be observed in 2 patients during breastfeeding. Hence, they were advised to suspend breastfeeding on the affected side. Besides, 1 patient stopped breastfeeding for 1 day. After no pus was found during breastfeeding the next day, breastfeeding was resumed. Another patient stopped breastfeeding for 3 days, which was resumed after no pus and blood fluid was discharged 4 days after treatment. There was no adverse reaction in the infants.

**Table 8:** Breastfeeding.

Type of disease	No. of cases	No. of continued breastfeeding cases (cases)	No. of days to suspend breast feeding on the affected breast (days)
Mastitis	38	38	0
Mammary abscess	3	3	4

### 3.8. Imaging changes under B ultrasound

Before treatment, 14 patients showed lamellar hypoechoic or anechoic areas of the breast with a maximum of 4.2 cm * 2.2 cm on B-ultrasound. Therefore, mastitis or breast abscess can be considered for these patients. Among them, 13 patients had normal B-ultrasound images after treatment. Besides, 1 patient had no obvious change and was hospitalized with intravenous administration of antibiotics and fine needle puncture under the guidance of B-ultrasound. The imaging recovery rate was 92.86%.

### 3.9. Cure rate

Among these 41 patients, 40 patients were clinically cured. After treatment, there was no breast pain, fear of cold, or fever in these patients. Besides, the breast lump and skin rash also disappeared. There was no side effect in these patients and no adverse reaction in the infants. The cure rate was 97.56%. Only 1 patient had no obvious mitigation in clinical symptoms after 5 days of treatment, and she was admitted to the hospital for further treatment (i.e., the patient with no obvious changes in imaging in Section 2.8).

## 4. Discussion

Acute mastitis during lactation is a kind of cellulitis involving the interlobular connective tissue in the breast. This disease usually occurs 6 weeks after delivery, but can also occur at any time point during lactation.^[9]^ As defined by WHO, acute mastitis during lactation is “a state of breast inflammation, which may or may not be accompanied by infection,”^[10,11]^ and it may lead to breast abscess and sepsis^[12]^ and directly induce cessation of breastfeeding. This disease is related to fatigue, pressure, breast duct blockage, insufficient feeding times, infant oral abnormalities (such as cleft lip or palate), local milk stasis, maternal malnutrition, primipara, use of manual breast suction device, community-acquired infection, breast damage, and poor diet.^[13]^ Further, it is usually caused by staphylococcus, streptococcus and/or corynebacterium infection.^[14]^ Acute mastitis during lactation may develop into breast abscesses in about 0.4% to 11% of patients.^[15]^ Patients with breast abscess are prone to a cessation of breastfeeding (41%), breast fistula (11.1%), and readmission (50%),^[3]^ which seriously affects the health of mothers and infants and increases the cost of health services. For example, the treatment cost of postpartum breast abscess in the United States varies from US $2340 to US $4012, which brings a huge economic burden to the medical system.^[3]^ Therefore, it is of great significance to identify an economical, convenient, effective, and pain-free treatment for these patients.

In the previous, empirical antibiotics were often administered orally or intravenously in the treatment of acute mastitis during lactation. However, the clinical efficacy is not satisfactory, due to the fact that many pathogens would generate antibiotic resistance after several treatment cycles.^[16]^ In this study, it was found that these pathogens were resistant to penicillin and cephalosporins in the milk bacterial culture and drug sensitivity tests for patients with acute mastitis and breast abscess during lactation. Therefore, antibiotics cannot achieve favorable efficacy in clinical practice. Patients with breast abscess during lactation are usually treated by antibiotics, incision and drainage, or ultrasound-guided puncture and aspiration. Nevertheless, there is no consensus on the optimal treatment method.^[17]^ However, there are some side effects in incision and drainage or ultrasound-guided puncture and aspiration, such as the influence of anesthesia on milk, separation of mother and infants, high cost, trauma and pain in patients, cessation of breastfeeding, and local scars on the breast.^[18]^ Therefore, there is an urgent demand for other effective treatment methods. In this context, it is worth making explorations into herbal medicines and TCM-based treatment.

In this study, Gualou Xiaoyong Decoction combined with painless lactation manipulation developed by our department was adopted in the treatment of patients with lactational mastitis and breast abscess. This combined therapy was verified to achieve favorable clinical efficacy. Among these 41 patients, 40 patients achieved clinical cure (the cure rate = 97.56%). After treatment, the clinical symptoms of these patients were alleviated significantly, and most laboratory indicators returned to a normal range. The pathogenic bacteria in the milk of most patients disappeared, and there was no abnormality in the B ultrasound imaging examination. Only 1 patient with breast abscess, since the clinical effect was not satisfactory after 5 days of treatment, she was admitted to the hospital to receive combined treatment for oral administration of Gualou Xiaoyong Decoction, painless lactation manipulation, intravenous administration of antibiotics, and fine needle aspiration under the guidance of B-ultrasound. This patient was discharged after 1 week.

During milk bacterial culture, it was found that there were diverse pathogenic bacteria in the milk. This indicated that this disease can be infected by 1 bacterium alone or by multiple bacteria together. Staphylococcus was the main pathogen. However, the pathogenic staphylococcus family contained many members, including *Staphylococcus aureus, Staphylococcus epidermidis, Staphylococcus warneri, Staphylococcus tardus*, and *Staphylococcus hominis*. In addition, there were other streptococcus families in the bacterial culture, such as Viridans streptococcus and salivary streptococcus. Moreover, it was revealed that the types of bacteria in the milk may change after treatment. Specifically, the culture results of most bacteria in the milk became negative after Gualou Xiaoyong Decotion in combination with painless lactation manipulation treatment. In this study, 3 patients had no abnormal bacterial culture results before treatment, but they developed Staphylococcus epidermidis after treatment. We speculated that it might be related to the progression of this disease, which may develop from milk stasis to noninfectious mastitis to infectious mastitis and then to breast abscess.^[9]^

In this study, 24 patients had negative bacterial culture results before treatment, but they had typical clinical symptoms of acute mastitis. Among them, 7 patients were able to cultivate bacteria from milk despite complete disappearance of clinical symptoms. However, bacteria can still be detected in the milk of 1 patient after clinical symptoms disappeared and the blood routine test, CRP, PCT, and B-ultrasound results returned to a normal range (*Staphylococcus aureus* and *Staphylococcus epidermidis* appeared alternately) (Case No. 35). After 45 days of treatment, the oral administration of herbal medicines was discontinued in this patient for follow-up observation, which continued for 2 months. Eventually, no signs of disease recurrence can be found. Additionally, there was also no abnormality in the infant. Therefore, it can be speculated that the type and number of pathogenic bacteria in the milk may be inconsistent with the severity of this disease. Some scholars also reported that there was an inconsistency between the bacterial load and the severity of lactational mastitis and breast abscess. Compared with the infection itself, the response of the immune system to injury may be highly related to the severity of the disease, and the bacteria themselves were not enough to induce the disease.^[19,20]^ In addition, mastitis is often manifested as inflammation lacking rich pathogenic bacteria, based on which some clinical researchers have described the “infectious” and “noninfectious” forms of mastitis.^[21]^ Pathogenic bacteria cannot be detected in the milk of many patients with mastitis.^[3]^ In view of this evidence, it is not surprising that the uncertainty about the administration of antimicrobial agents in the treatment of mastitis has been highlighted in recent Cochrane systematic reviews. Some scholars have proposed that the main driving factor of mastitis is the inflammatory mediators in the host, and these same inflammatory mediators may also lead to insufficient lactation in clinical and subclinical mastitis.^[6]^

All patients were encouraged to persist in breastfeeding. In this study, there were 2 patients with breast abscess who stopped breastfeeding on the affected breast for 1 day and 3 days respectively after the occurrence of pus during lactation. Apart from these 2 patients, other patients did not stop breastfeeding, and no adverse reaction was observed in the infants. As per a WHO report, breastfeeding shall be resumed immediately after the symptoms of the affected breast are alleviated, so as to prevent milk stasis and recurrence of infection.^[22]^ Of note, the mouth of the infant shall not contact the infected fluid or breast tissue.^[9]^ If breastfeeding cannot be resumed in time, viscous liquid may be generated, which will aggravate milk stasis. Therefore, breastfeeding ensures the drainage of affected areas and rapid resolution of abscesses.^[17]^ Similarly, the Program Committee of the International Society of Breastfeeding Medicine also maintains that there is no evidence to prove that infected breastfeeding can pose a threat to infant health. We also believe that resuming breastfeeding can reduce milk stasis, which is conducive to disease recovery, and infants can get enough nutrition from milk. Further, no adverse effects were found in infants during resuming breastfeeding in clinical practice.

Gualou Xiaoyong Decoction is a mature prescription developed by our department. It can achieve favorable clinical effects in the treatment of lactational acute mastitis and breast abscess. Chaihu and Gualou peel are used to soothe the liver and regulate qi; Stir fry Atractylodes macrocephala with bran can strengthen the spleen and stomach; *Arctium lappa* L., *Scutellaria barbata* L., dandelion, honeysuckle, forsythia suspensa L can be used to clear away heat and toxic materials, remove stasis, and subside swelling; Angelica, Paeonia lactiflora, and Peach Kernel can be used to cool the blood, nourish the blood, and activate blood circulation to dissipate blood stasis; Coix seed and adzuki bean can be used to detoxify and discharge pus; Luffa and grass can be used to activate meridians, remove stasis, and realize lactogenesis. The whole prescription has the effects of soothing the liver and stomach, resolving phlegm, removing heat, relieving internal toxic materials, releasing pus, and realizing lactogenesis.

The accumulation of milk is also an important factor causing mastitis during lactation. The accumulated milk not only causes breast pain and discomfort and reduces milk volume, but also provides an ideal bacterial culture medium, which causes the aggravation and recurrence of breast inflammation.^[7]^ Therefore, it is very important to empty the breast. The painless lactation technique developed by our department could achieve favorable efficacy, and it would not cause pain and side effects in patients. This technique mainly combines the massage of distant acupoints along the meridian with local breast massage, and hence it can achieve a favorable effect on lactation. Through this combined therapy, the clinical symptoms of patients with lactational acute mastitis can be significantly alleviated in a short period. Therefore, this method is expected to become an effective therapy in the treatment of this disease, and hence it is worthy of clinical promotion. Meanwhile, this study has some limitations. This project is a retrospective study of cases, but there is still a lack of prospective cohort study and control study. Next, we will do some prospective study, such as a comparative study of Gualou Xiaoyong Decoction in combination with painless breast feeding manipulation, and western cephalosporins or penicillin antibiotics orally or intravenous drip, to further understand the advantages of Chinese medicine treatment.

## Acknowledgments

This paper was completed under the careful guidance and strong support of Dr Jin. Dr Jin has had an important impact on me with his rigorous and realistic academic attitude, high professionalism, conscientious, diligent work style and innovative enterprising spirit. Her profound knowledge, broad vision and sharp thinking gave me deep inspiration. At the same time, during the design of this paper, I also learned a lot about Chinese medicine, and my clinical skills have been greatly improved.

## Author contributions

**Investigation:** Huijun Ye.

**Resources:** Huiling Zheng.

**Supervision:** Lihua Jin.

**Writing – original draft:** Dexin Hu, Yi Yang, Xi Luo.

**Writing – review & editing:** Yunxia Lin, Jiali Liu, Ruilan Li, Fengying Hu, Lihua Jin.
